# Deep learning in rib fracture imaging: study quality assessment using the Must AI Criteria-10 (MAIC-10) checklist for artificial intelligence in medical imaging

**DOI:** 10.1186/s13244-025-02046-x

**Published:** 2025-08-09

**Authors:** Jonas M. Getzmann, Kitija Nulle, Cinzia Mennini, Umberto Viglino, Francesca Serpi, Domenico Albano, Carmelo Messina, Stefano Fusco, Salvatore Gitto, Luca Maria Sconfienza

**Affiliations:** 1 IRCCS Istituto Ortopedico Galeazzi, Milan, Italy; 2 Radiology Department, Riga East Clinical University Hospital, Riga, Latvia; 3 Unit of Radiology, Ospedale Evangelico Internazionale, Genoa, Italy; 4 Dipartimento di Scienze Biomediche, Chirurgiche ed Odontoiatriche, Università degli Studi di Milano, Milan, Italy; 5 UOC Radiodiagnostica, ASST Centro Specialistico Ortopedico Traumatologico Gaetano Pini-CTO, Milan, Italy; 6 Dipartimento di Scienze Biomediche per la Salute, Università degli Studi di Milano, Milan, Italy

**Keywords:** Artificial intelligence, Deep learning, Checklist, Guideline, Fracture

## Abstract

**Objectives:**

To analyze the methodological quality of studies on deep learning (DL) in rib fracture imaging with the Must AI Criteria-10 (MAIC-10) checklist, and to report insights and experiences regarding the applicability of the MAIC-10 checklist.

**Materials and methods:**

An electronic literature search was conducted on the PubMed database. After selection of articles, three radiologists independently rated the articles according to MAIC-10. Differences of the MAIC-10 score for each checklist item were assessed using the Fleiss’ kappa coefficient.

**Results:**

A total of 25 original articles discussing DL applications in rib fracture imaging were identified. Most studies focused on fracture detection (*n* = 21, 84%). In most of the research papers, internal cross-validation of the dataset was performed (*n* = 16, 64%), while only six studies (24%) conducted external validation. The mean MAIC-10 score of the 25 studies was 5.63 (SD, 1.84; range 1–8), with the item “clinical need” being reported most consistently (100%) and the item “study design” being most frequently reported incompletely (94.8%). The average inter-rater agreement for the MAIC-10 score was 0.771.

**Conclusions:**

The MAIC-10 checklist is a valid tool for assessing the quality of AI research in medical imaging with good inter-rater agreement. With regard to rib fracture imaging, items such as “study design”, “explainability”, and “transparency” were often not comprehensively addressed.

**Critical relevance statement:**

AI in medical imaging has become increasingly common. Therefore, quality control systems of published literature such as the MAIC-10 checklist are needed to ensure high quality research output.

**Key Points:**

- Quality control systems are needed for research on AI in medical imaging.
- The MAIC-10 checklist is a valid tool to assess AI in medical imaging research quality.
- Checklist items such as “study design”, “explainability”, and “transparency” are frequently addressed incomprehensively.

**Graphical Abstract:**

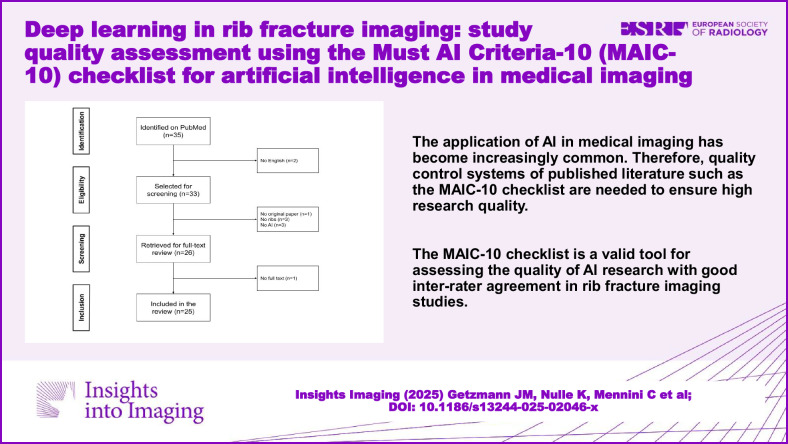

## Introduction

Artificial intelligence (AI)-based technologies dominate medical headlines and promise to revolutionize medicine globally [1]. High rates of medical errors, clinical workflow inefficiencies, and unsustainable utilization of resources may potentially be overcome with the correct use of AI. In addition, the amount of research in which AI-based technologies are making important contributions towards faster and more accurate diagnosis, and therefore reliable prognosis, is growing [2]. At the same time, the number of proposed frameworks for ensuring the safety and effectiveness of AI-based software as a medical device is also increasing [3]. To ensure appropriate methodological quality control mechanisms, numerous sets of criteria have been proposed, and one of the latest guidelines published to assess AI in medical imaging is the Must AI Criteria-10 (MAIC-10) checklist [4]. The goal of the MAIC-10 checklist is to provide a concise definition of essential core standards to which every publication on AI-based technologies in medical imaging should adhere. It includes several items, as described in Table 1. These include: (1) clinical need—justification of the clinical relevance; (2) study design—appropriate and transparent design strategy; (3) safety and privacy—protection of patient data; (4) data curation—proper handling and quality assurance of datasets; (5) data annotation—accuracy and reproducibility of labeled data; (6) data partitioning—transparency in training, validation, and testing splits; (7) AI model—comprehensive description of the model architecture and implementation; (8) robustness—assessment of model performance stability; (9) explainability—clarity in model decision pathways; and (10) transparency—openness of methodology, including code and data availability.

**Table 1:** MAIC-10 checklist to assess quality of AI-based medical imaging research studies (adopted from [4])

Checklist item	Article section	Description
1. Clinical need	Introduction	The study is clearly put into context by describing the target clinical problem and any previous approaches in the literature
2. Study design	Materials and methods	The type of study (observational/interventional, single/multicenter) and inclusion/exclusion criteria are explicitly described, and a sample size estimate is given
3. Safety and privacy	Materials and methods	ELSI (ethical, legal, and social implications), specifically including ethics committee approval and data de‐identification issues, are discussed
4. Data curation	Materials and methods	Data extraction, cleaning, and transformation methods, including image pre‐processing steps, are clearly described
5. Data annotation	Materials and methods	The ground truth reference is defined, and the annotation process, including measures of inter/intra-observer variability, is described
6. Data partitioning	Materials and methods	Methods and criteria for data set splitting into train‐tune‐test‐validation sets are indicated
7. AI model	Materials and methods, results	The AI model building methodology is sufficiently detailed by including used technologies (software and hardware), training–tuning–testing methods, performance metrics, and resulting AI model architecture
8. Robustness	Results, discussion	The generalizability of the AI model in real‐world conditions is explicitly discussed
9. Explainability	Discussion	The interpretability of the model (including the use of uncertainty or confidence metrics) is explicitly discussed
10. Transparency	Discussion	Any possibility of access to original data sets and source code is clearly stated. Financing and conflicts of interest are detailed

All the listed sub-items under the descriptions should be addressed to consider that the corresponding item has been fulfilled

In this study, we investigated the applicability of the MAIC-10 checklist in studies on the use of AI in rib fracture imaging. Rib fractures are the most common type of chest injury [5]. Depending on the number of ribs fractured, bone fragment dislocation, or damage to the surrounding structures, rib fractures are associated with complications and can impact the choice of therapy and outcome after injury [6]. Several imaging studies have recently focused on AI, particularly deep learning (DL), and rib fractures with the aim of ensuring accurate diagnosis and, consequently, improving prognosis.

Thus, the objectives of this study were to analyze the methodological quality of studies on DL in rib fracture imaging with MAIC-10, and to report insights and experiences regarding the applicability of the MAIC-10 checklist.

## Methods

### Literature search and study selection

Institutional Review Board approval was not needed for this study, which consisted of an analysis of published literature, and no patients were involved. An electronic literature search was conducted by two independent reviewers (J.M.G. and K.N.) on the PubMed database for articles published up to August 31, 2024. The search query was performed using the following keywords and their expansions: (“rib fracture”) AND (“artificial intelligence” OR “deep learning” OR “neural networks” OR “predictive models”). The reviewers assessed potential studies by screening titles and abstracts. Research articles were included if they met the following criteria: (i) the use of AI in either diagnostic or prognostic purposes with regard to rib fractures; (ii) English language; and (iii) statement that approval from the local ethics committee and informed consent from each patient or a waiver for it was obtained (if this information was not available in the abstract, the full text was reviewed to confirm eligibility).

The exclusion criteria were (i) studies reporting insufficient data for outcomes (insufficient data reporting was defined as missing critical methodological or results-related information that prevented a sufficient quality assessment using MAIC-10); (ii) reviews, guidelines, consensus statements, editorials, letters, comments, or conference abstracts.

Articles that met the inclusion criteria were obtained in full, including any supplementary material. The eligibility was further determined based on the full-text articles by the reviewers. The reference lists of included articles were scanned for further potentially eligible studies.

### Data extraction and quality assessment

Three radiologists (K.N., C.M., and U.V.) independently rated the articles in accordance with the MAIC-10 checklist. A training phase was introduced to prepare the three readers for the assessment of the 25 articles. This was performed using a research article, which was not included in the final list of papers to assess for the current study. The review of the training study using the MAIC-10 checklist was discussed until the three readers understood each parameter. If they could not come to an agreement, a fourth radiologist with five years of experience in AI research (S.G.) was asked to join the discussion in order to reach a conclusion.

### Data analysis

All statistical analyses were conducted using SPSS (version 29.0.1.0; IBM). *p* values < 0.05 were considered statistically significant. Differences of the MAIC-10 score for each checklist item were assessed using the Fleiss’ kappa coefficient. The strength of the Fleiss’ kappa is based on the values of Cohen’s kappa coefficient [7]. Fleiss’ kappa coefficient of 0.20 or less is considered poor, 0.21–0.40 fair, 0.41–0.60 moderate, 0.61–0.80 good, and 0.80–1.00 very good [7, 8].

## Results

### Literature search

**Fig. 1:**
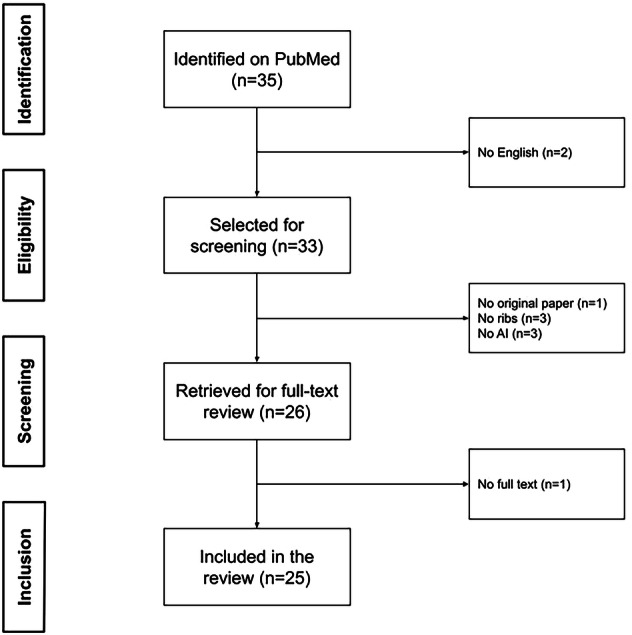
Flowchart of systematic identification, screening, eligibility, and inclusion information from retrieved studies. AI, artificial intelligence

A flowchart illustrating the literature search process is presented in Fig. 1. The electronic literature search resulted in 35 articles from PubMed. Two studies were not available in English and were excluded. A total of 33 abstracts were screened. Among these, one study was a literature review, three studies did not include imaging of the ribs, and three studies did not include AI. For one study, only an abstract was available. After applying all the eligibility criteria, 25 studies were included.

### Baseline study characteristics

Table 2 describes the baseline study characteristics of the included studies. Most of the articles were published between 2020 and 2023 (*n* = 24, 96%). Only one paper was published before that in 1995 [9]. The mean sample size was 1130 (standard deviation (SD), 4188; range, 39–20,260).

**Table 2:** Baseline study characteristics of papers dealing with artificial intelligence in rib fracture imaging

First author	Year	Number of patients (*n*)	Study topic	Intended use	Imaging type	Validation type
Azuma [22]	2022	463	Fracture detection	Diagnostic	CT	Internal
Castro-Zunti [5]	2021	612	Fracture detection	Diagnostic	CT	Internal
Choi J [10]	2022	20’260	Prediction of outcome (readmission)	Prognostic	CT	Internal
Choi J [11]	2022	332	Detection of pulmonary contusion	Diagnostic	CT	Internal
Dombi [9]	1995	580	Prediction of outcome	Prognostic	CT	Internal
Gao [23]	2022	600	Fracture detection	Diagnostic	CT	Internal
Gipson [13]	2022	1404	Fracture detection	Diagnostic	X-ray	n/a
Guermazi [24]	2022	480	Fracture detection	Diagnostic	X-ray	Internal
Hongbiao [14]	2023	123	Fracture detection	Diagnostic	CT	n/a
Ibanez [25]	2022	195	Fracture detection	Diagnostic	CT	Internal
Jin [26]	2020	900	Fracture detection	Diagnostic	CT	Internal
Kaiume [15]	2021	39	Fracture detection	Diagnostic	CT	n/a
Kaviani [12]	2022	279	Chest X-ray findings	Diagnostic	X-ray	External
Liu [17]	2022	393	Fracture detection	Diagnostic	CT	Internal
Meng [27]	2021	8529	Fracture detection	Diagnostic	CT	External
Niiya [28]	2022	656	Fracture detection	Diagnostic	CT	Internal
Weikert [29]	2020	511	Fracture detection	Diagnostic	CT	Internal
Wu [30]	2023	1080	Fracture detection	Diagnostic	X-ray	External
Yang [6]	2022	591	Fracture detection	Diagnostic	CT	External
Yao [31]	2021	1707	Fracture detection	Diagnostic	CT	Internal
Zhang [32]	2021	102	Fracture detection	Diagnostic	CT	Internal
Zhou QQ [33]	2022	640	Fracture detection	Diagnostic	CT	External
Zhou QQ [34]	2021	1020	Fracture detection	Diagnostic	CT	Internal
Zhou QQ [35]	2020	1079	Fracture detection	Diagnostic	CT	External
Zhou Z [36]	2022	818	Fracture detection	Diagnostic	CT	Internal

Several research topics were covered in the included articles. Among them, most studies focused on rib fracture detection (*n* = 21, 84%). Only two studies (8%) focused on the prediction of injury outcomes [9, 10], while one study (4%) focused on the detection of pulmonary contusions [11] and another one (4%) on specific findings on chest X-rays [12]. The DL approach was most frequently used as a diagnostic tool (*n* = 23, 92%), and in two articles (8%) as a prognostic tool [9, 10]. In terms of modalities, 21 studies (84%) used CT and four studies (16%) used X-ray. In most of the research papers, internal cross-validation of the dataset was performed (*n* = 16, 64%), while only six studies (24%) conducted external validation. Three articles (12%) did not report on validation strategies [13–15].

### MAIC-10 adherence

The items of the MAIC-10 checklist are shown in Table 1. The mean MAIC-10 score of the 25 studies was 5.63 (SD, 1.84; range 1–8), which was 56.3% of the ideal score of 10. The adherence to individual MAIC-10 items is described based on the mean score of all raters.

The checklist item “clinical need” was addressed in all studies (*n* = 25, 100%). Only 5.2% of studies reported on all aspects of “study design”. The item ‘’safety and privacy” of the MAIC-10 checklist was properly addressed in 37.3% of studies. ‘’Data curation”, ‘’data annotation” and ‘’data partitioning” were properly and completely described in 69.3%, 74.6%, and 68.0% of articles, respectively. 61.3% of the studies provided full information about the “AI model”. 74.6% of articles addressed the ‘’robustness” of the research. ‘’Explainability” and ‘’transparency” were correctly addressed in 36.0% and 34.6%, respectively.

The inter-rater agreement of MAIC-10 was calculated (Table 3). The average inter-rater agreement for the MAIC-10 score was 0.771. Specifically, very good agreement was achieved in evaluating the items ‘’clinical need” (*K* = 1.000), ‘’study type” (*K* = 0.930), ‘’data curation” (*K* = 0.875), “data annotation” (*K* = 0.930), and ‘’transparency” (*K* = 0.823). Poor agreement was obtained only in evaluating the item “explainability” (*K* = 0.190).

**Table 3:** Adherence to individual items of the MAIC-10 checklist and inter-rater agreement

MAIC-10 item	Kappa	95% CI lower limit	95% CI upper limit
Clinical need	1.000	1.000	1.000
Study type	0.930	0.703	1.000
Safety and privacy	0.772	0.546	0.998
Data curation	0.875	0.648	1.000
Data annotation	0.930	0.703	1.000
Data partitioning	0.755	0.529	0.981
AI model	0.719	0.493	0.945
Robustness	0.718	0.492	0.944
Explainability	0.190	−0.037	0.416
Transparency	0.823	0.597	1.000

*CI* confidence interval, *Kappa* Fleiss’ kappa coefficient, *MAIC-10* Must AI Criteria-10 checklist

## Discussion

The role of AI-based technologies in rib fracture imaging has demonstrated remarkable growth, mainly due to the advances of computers and software solutions. However, AI-based solutions require strict quality control before introduction into routine clinical practice. Furthermore, AI developments have come so far that it is not enough for the model to perform well, it is paramount that we understand why the model makes a certain decision [16]. To guarantee this transparency and explainability, numerous authors have come up with criteria they consider essential to guarantee minimum quality standards.

After reviewing a selection of articles on AI in rib fracture imaging, we tested the applicability of the MAIC-10 checklist. MAIC-10 seemed to be a valid tool for the quality assessment of the selected articles, with good inter-rater agreement. However, some items of the checklist were often not comprehensively addressed.

The mean MAIC-10 score of all reviewed publications was 5.63 out of 10. This relatively low mean score might be explained by the use of sub-items in the checklist that add a level of complexity to it. If one study fails to mention only one of the sub-items, it would not obtain a point for the respective checklist item. An alternative approach could involve weighting checklist sub-items based on their impact on study reliability, or introducing tiered scoring rather than binary assessment.

With regard to the different items of the checklist, all studies addressed the clinical background and described previous approaches in the literature, and the intended use and role of the AI application (“clinical need”). The second item of the checklist (“study design”) was, however, not comprehensively addressed in most of the studies. Although most papers described the methodology in a clear manner, only the study by Liu et al [17] also provided a sample size estimate and related calculations. Inadequate reporting or unacknowledged changes in sample size can introduce bias and lead to misinterpretation of results [18].

Most of the articles included in our analysis mentioned that the data was anonymized. However, a detailed description of the anonymization with regard to data pre-processing, as well as the description of the de-identification protocol, was found to be often missing. This was not in accordance with the MAIC-10 checklist, which actively emphasizes the importance of transparent reporting, data protection protocols, and easy-to-reproduce de-identification (“safety and privacy”).

“Data curation”, “data annotation”, and “data partitioning” were relatively well described in the selected articles on rib imaging (in 69.3%, 74.6%, and 68.0% of the articles, respectively). Sub-items that were frequently missing were the reasoning for data set splitting and train-test-validation of data set divisions in “data partitioning”. 61.3% of the articles provided full information about the “AI model,” including software and hardware, training-tuning-testing methods, and performance metrics. The sub-item that was most frequently incompletely described was hardware. Even when hardware was mentioned, the extent and details of the information provided varied greatly.

“Robustness” was addressed in 74.6% of the articles, however depth of details and provided analysis varied as well. The concept of “robustness” as captured in the MAIC-10 checklist does not explicitly require the assessment of model performance across different demographic groups or imaging devices. This raises the concern that important aspects of external validity and generalizability may be underrepresented in current evaluations. Given the increasing deployment of AI models in diverse clinical settings, it may be warranted to extend the MAIC-10 checklist by incorporating a dedicated item focused on dataset diversity and external applicability.

“Explainability” and “transparency” were the two items of the checklist that were most frequently described inconsistently in the selected articles on rib imaging (i.e., addressed correctly in only 36,0% and 34,6% of papers, respectively). Common barriers to transparency include proprietary AI models and a lack of open-access datasets. Furthermore, low inter-rater agreement was found for the item “explainability”. One of the reasons might be that this item of the checklist was perceived as subjective by the readers and therefore showed greater interpretability. One possible solution to standardize interpretation would be the addition of accompanying examples to the checklist. On the other hand, it is generally important that the explanation of the AI model is as clear as possible in order to avoid the “black box effect” (i.e., poor explainability) and ensure that the study’s methodology is described so clearly that others can reproduce the experiment. Future studies should incorporate standardized methods whenever possible and provide transparency by sharing model architectures and training protocols in detail.

There are several limitations in this study. First, the authors only included AI-based articles in rib fracture imaging, not involving other medical sub-disciplines. While this may limit generalizability, it was a deliberate design choice to assess MAIC-10 within a well-defined medical imaging application. Second, an electronic literature search was performed in the PubMed database only. Third, the evaluation of included studies was performed by radiologists only, which might introduce a certain bias. Studies utilizing AI-based technologies are usually conducted by multidisciplinary teams [19–21]. Hence, it might be challenging for a single reader to comprehend all aspects of the study, which could in turn lead to possible under- or overscoring due to variable interpretation of checklist items. Fourth, the number of articles included in this study was relatively small (*n* = 25), and a great heterogeneity in terms of objectives and evaluated parameters was seen.

In conclusion, after reviewing selected articles on rib fracture imaging according to MAIC-10, some quality aspects have not been comprehensively addressed. However, during the evaluation process, questions about the clarity of the checklist items themselves have arisen. It is evident that profound knowledge of statistics and AI-based solutions is necessary not only to thoroughly understand the research, but also the checklist itself and the reasoning behind it. Nevertheless, the inevitable tendency of AI to emerge in medical imaging and increasingly also in statistical and technical studies requires implementing quality criteria. The MAIC-10 checklist seems to be a valid evaluation tool for assessing the quality of such studies.

## Data Availability

Datasets including extracted and analyzed data can be obtained upon request by sending an email to the corresponding author.

## Abbreviations

AI: Artificial intelligence
DL: Deep learning
MAIC-10: Must AI Criteria-10
SD: Standard deviation

## References

[1] Gitto S, Serpi F, Albano D et al (2024) AI applications in musculoskeletal imaging: a narrative review. Eur Radiol Exp 8:22 38355767 10.1186/s41747-024-00422-8 PMC10866817

[2] Alowais SA, Alghamdi SS, Alsuhebany N et al (2023) Revolutionizing healthcare: the role of artificial intelligence in clinical practice. BMC Med Educ 23:689 37740191 10.1186/s12909-023-04698-z PMC10517477

[3] Sounderajah V, Normahani P, Aggarwal R et al (2022) Reporting standards and quality assessment tools in artificial intelligence-centered healthcare research. In: Lidströmer N, Ashrafian H (eds) Artificial intelligence in medicine. Springer, Cham

[4] Cerdá-Alberich L, Solana J, Mallol P et al (2023) MAIC-10 brief quality checklist for publications using artificial intelligence and medical images. Insights Imaging 14:11 36645542 10.1186/s13244-022-01355-9 PMC9842808

[5] Castro-Zunti R, Chae KJ, Choi Y, Jin GY, Ko SB (2021) Assessing the speed-accuracy trade-offs of popular convolutional neural networks for single-crop rib fracture classification. Comput Med Imaging Graph 91:101937 34087611 10.1016/j.compmedimag.2021.101937

[6] Yang C, Wang J, Xu J et al (2022) Development and assessment of deep learning system for the location and classification of rib fractures via computed tomography. Eur J Radiol 154:110434 35797792 10.1016/j.ejrad.2022.110434

[7] Gwet KL (2021) Large-sample variance of Fleiss generalized Kappa. Educ Psychol Meas 81:781–790 34267400 10.1177/0013164420973080 PMC8243202

[8] Si L, Zhong J, Huo J et al (2021) Deep learning in knee imaging: a systematic review utilizing a checklist for artificial intelligence in medical imaging (CLAIM). Eur Radiol 32:1353–1361 34347157 10.1007/s00330-021-08190-4

[9] Dombi GW, Nandi P, Saxe JM, Ledgerwood AM, Lucas CE (1995) Prediction of rib fracture injury outcome by an artificial neural network. J Trauma 39:915–921 7474008 10.1097/00005373-199511000-00016

[10] Choi J, Alawa J, Tennakoon L, Forrester JD (2022) DeepBackRib: deep learning to understand factors associated with readmissions after rib fractures. J Trauma Acute Care Surg 93:757–761 36121263 10.1097/TA.0000000000003791

[11] Choi J, Mavrommati K, Li NY et al (2022) Scalable deep learning algorithm to compute percent pulmonary contusion among patients with rib fractures. J Trauma Acute Care Surg 93:461–466 35319542 10.1097/TA.0000000000003619

[12] Kaviani P, Digumarthy SR, Bizzo BC et al (2022) Performance of a chest radiography AI algorithm for detection of missed or mislabeled findings: a multicenter study. Diagnostics 12:2086 36140488 10.3390/diagnostics12092086 PMC9497851

[13] Gipson J, Tang V, Seah J et al (2022) Diagnostic accuracy of a commercially available deep-learning algorithm in supine chest radiographs following trauma. Br J Radiol 95:20210979 35271382 10.1259/bjr.20210979 PMC10996416

[14] Hongbiao S, Shaochun X, Xiang W et al (2023) Comparison and verification of two deep learning models for the detection of chest CT rib fractures. Acta Radiol 64:542–551 35300519 10.1177/02841851221083519

[15] Kaiume M, Suzuki S, Yasaka K et al (2021) Rib fracture detection in computed tomography images using deep convolutional neural networks. Medicine (Baltimore) 100:e26024 34011107 10.1097/MD.0000000000026024 PMC8137061

[16] Doshi-Velez F, Kim B (2017) Towards A rigorous science of interpretable machine learning. Preprint at 10.48550/arXiv.1702.08608

[17] Liu X, Wu D, Xie H et al (2022) Clinical evaluation of AI software for rib fracture detection and its impact on junior radiologist performance. Acta Radiol 63:1535–1545 34617809 10.1177/02841851211043839

[18] Chan AW, Hróbjartsson A, Jørgensen KJ, Gøtzsche PC, Altman DG (2008) Discrepancies in sample size calculations and data analyses reported in randomised trials: comparison of publications with protocols. BMJ 337:a2299 19056791 10.1136/bmj.a2299 PMC2600604

[19] Gitto S, Cuocolo R, Huisman M et al (2024) CT and MRI radiomics of bone and soft-tissue sarcomas: an updated systematic review of reproducibility and validation strategies. Insights Imaging 15:54 38411750 10.1186/s13244-024-01614-x PMC10899555

[20] Gitto S, Cuocolo R, Albano D et al (2021) CT and MRI radiomics of bone and soft-tissue sarcomas: a systematic review of reproducibility and validation strategies. Insights Imaging 12:68 34076740 10.1186/s13244-021-01008-3 PMC8172744

[21] Gitto S, Annovazzi A, Nulle K et al (2024) X-rays radiomics-based machine learning classification of atypical cartilaginous tumour and high-grade chondrosarcoma of long bones. EBioMedicine 101:105018 38377797 10.1016/j.ebiom.2024.105018 PMC10884340

[22] Azuma M, Nakada H, Takei M et al (2022) Detection of acute rib fractures on CT images with convolutional neural networks: effect of location and type of fracture and reader’s experience. Emerg Radiol 29:317–328 34855002 10.1007/s10140-021-02000-6

[23] Gao Y, Chen H, Ge R et al (2022) Deep learning-based framework for segmentation of multiclass rib fractures in CT utilizing a multi-angle projection network. Int J Comput Assist Radiol Surg 17:1115–1124 35384552 10.1007/s11548-022-02607-1

[24] Guermazi A, Tannoury C, Kompel AJ et al (2021) Improving radiographic fracture recognition performance and efficiency using artificial intelligence. Radiology 302:627–636 34931859 10.1148/radiol.210937

[25] Ibanez V, Gunz S, Erne S et al (2022) RiFNet: automated rib fracture detection in postmortem computed tomography. Forensic Sci Med Pathol 18:20–29 34709561 10.1007/s12024-021-00431-8 PMC8921053

[26] Jin L, Yang J, Kuang K et al (2020) Deep-learning-assisted detection and segmentation of rib fractures from CT scans: Development and validation of FracNet. EBioMedicine 62:103106 33186809 10.1016/j.ebiom.2020.103106 PMC7670192

[27] Meng XH, Wu DJ, Wang Z et al (2021) A fully automated rib fracture detection system on chest CT images and its impact on radiologist performance. Skeletal Radiol 50:1821–1828 33599801 10.1007/s00256-021-03709-8

[28] Niiya A, Murakami K, Kobayashi R et al (2022) Development of an artificial intelligence-assisted computed tomography diagnosis technology for rib fracture and evaluation of its clinical usefulness. Sci Rep 12:8363 35589847 10.1038/s41598-022-12453-5 PMC9119970

[29] Weikert T, Noordtzij LA, Bremerich J et al (2020) Assessment of a deep learning algorithm for the detection of rib fractures on whole-body trauma computed tomography. Korean J Radiol 21:891–899 32524789 10.3348/kjr.2019.0653 PMC7289702

[30] Wu J, Liu N, Li X et al (2023) Convolutional neural network for detecting rib fractures on chest radiographs: a feasibility study. BMC Med Imaging 23:18 36717773 10.1186/s12880-023-00975-x PMC9885575

[31] Yao L, Guan X, Song X et al (2021) Rib fracture detection system based on deep learning. Sci Rep 11:23513 34873241 10.1038/s41598-021-03002-7 PMC8648839

[32] Zhang B, Jia C, Wu R et al (2021) Improving rib fracture detection accuracy and reading efficiency with deep learning-based detection software: a clinical evaluation. Br J Radiol 94:20200870 33332979 10.1259/bjr.20200870 PMC7934317

[33] Zhou QQ, Hu ZC, Tang W et al (2022) Precise anatomical localization and classification of rib fractures on CT using a convolutional neural network. Clin Imaging 81:24–32 34598000 10.1016/j.clinimag.2021.09.010

[34] Zhou QQ, Tang W, Wang J et al (2021) Automatic detection and classification of rib fractures based on patients’ CT images and clinical information via convolutional neural network. Eur Radiol 31:3815–3825 33201278 10.1007/s00330-020-07418-z

[35] Zhou QQ, Wang J, Tang W et al (2020) Automatic detection and classification of rib fractures on thoracic CT using convolutional neural network: accuracy and feasibility. Korean J Radiol 21:869–879 32524787 10.3348/kjr.2019.0651 PMC7289688

[36] Zhou Z, Fu Z, Jia J, Lv J (2022) Rib fracture detection with dual-attention enhanced U-Net. Comput Math Methods Med 2022:8945423 36035283 10.1155/2022/8945423 PMC9410867

